# Supplementary material for: Anti-Inflammatory Polymeric Nanoparticles Based on Ketoprofen and Dexamethasone
Source: Pharmaceutics. 2020 Jul 31;12(8):723. doi: 10.3390/pharmaceutics12080723 (PMC7465001; doi:10.3390/pharmaceutics12080723)
Supplement: Supplementary file 1 [file pharmaceutics-12-00723-s001.pdf]

pharmaceutics

Supplementary Materials: Anti-Inflammatory Polymeric Nanoparticles Based on Ketoprofen and Dexamethasone

Eva Espinosa-Cano, Maria Rosa Aguilar, Yadileiny Portilla, Domingo F. Barber and Julio San Román

Materials and methods

Synthesis and characterization of copolymer of ketoprofen-based methacrylic monomer and 1-vinyl imidazole, poly(HKT-co-VI).

Determination of reactivity ratios. Monomers reactivity ratios were calculated through "in situ" ^{1}H-NMR monitorization of the copolymerization reaction following the second methodology described by Aguilar, M.R. et al. in 2002. [1] Briefly, copolymerization reactions (F_{HKT} = 20, 40 and, 60; [M] = 0.25 M and [AIBN] = 1.5 × 10^{-2} M) were carried out inside the NMR equipment (Varian Mercury 400 MHz) using deuterated DMSO (DMSO-$_{d6}$, Merck) as a solvent at 60 ºC, 90º pulse and acquiring a new spectrum each 120 seconds. A solution of DMF (10 mg/mL) in DMSO-d6 in a thin wall capillary tube introduced in the NMR tube was used as reference.

Preparation and characterization of self-assembled nanoparticles

Hydrodynamic properties. Nanoparticles were prepared in triplicates at pH = 4.0 and hydrodynamic properties were measured. pH was increased by 0.5 via the addition of 1 M NaOH aqueous solution and size distribution and ξ were measured. The procedure was repeated until the aggregation pH was reached. After that, the pH was set to 4.0 again to check reversibility of the aggregation phenomenon. Samples size distribution and ξ were measured every week up to one month to demonstrate NPs stability when stored in suspension at 4°C and at the most suitable storage pH. NPs were freeze-dried after preparation and dispersed in the same volume of 0.1 M acetic acid buffer solution. The suspension was then subjected to manual shaking and ultrasound bath/tip sonication (30% amplitude) for 10 minutes. After sonication, size distribution of NPs was measured to check redispersibility.

Scanning electron microscopy NPs morphology characterization. A SEM analysis of KT NPs was performed with a Hitachi SU8000 TED, cold-emission FE-SEM microscope working with an accelerating voltage 1 kV-D. Sample preparation consisted in the deposition of one drop of the corresponding NPs suspension (0.02 mg/mL) over a small glass disk (12 mm diameter) and evaporation at room temperature. Gold palladium alloy (80:20) was used to coat the samples prior to examination by SEM.

Results

NPs stabilization in culture media

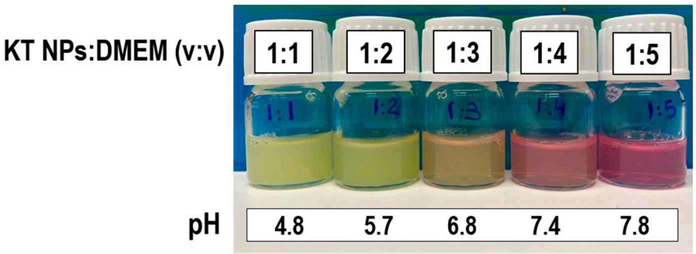

Figure S1. Color and pH values of nanoparticles (KT NPs, 3 mg/mL) in suspension at different NPs:DMEM volume ratios.

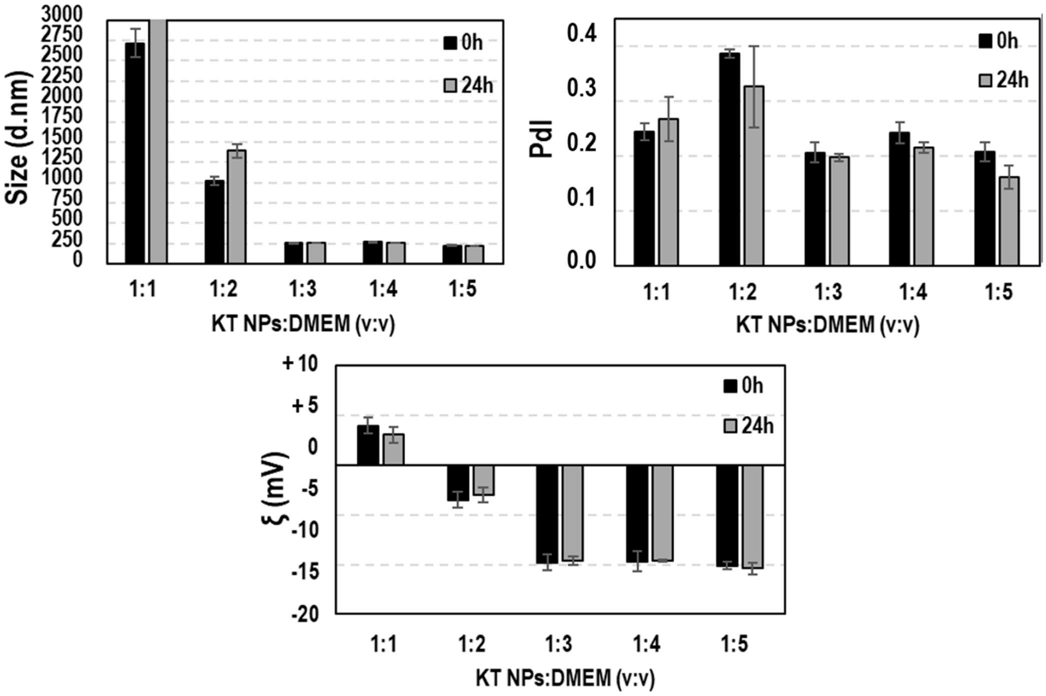

Figure S2. Hydrodynamic properties (i.e. diameter (size) in nanometers, polydispersity (PdI) and, zeta potential (ξ) in mV of nanoparticles (KT NPs, 3 mg/mL) in suspension at different NPs:DMEM volume ratios.

Determination of reactivity ratios

Reactivity ratios of the co-monomers (r_{HKT} and r_{VI}) were determined as previously described by Aguilar et al. [1]. $r_{HKT} = 3.74 \pm 1.20$ and $r_{VI} = 0.05 \pm 0.06$ meaning that active radicals incorporate HKT monomer around 100 times faster than VI. These values were consistent with previously reported copolymerization reactions between VI and methacrylic derivatives using AIBN as free radical initiator [2,3] and they revealed that the desired gradient microstructure was obtained. **Figure S1** shows the surface co-polymerization diagram representing the length of VI blocks as a function of conversion. The length of VI blocks increases with conversion and the higher the feed molar contents in HKT the higher the conversion degree needed to obtain longer hydrophilic VI-enriched segments.

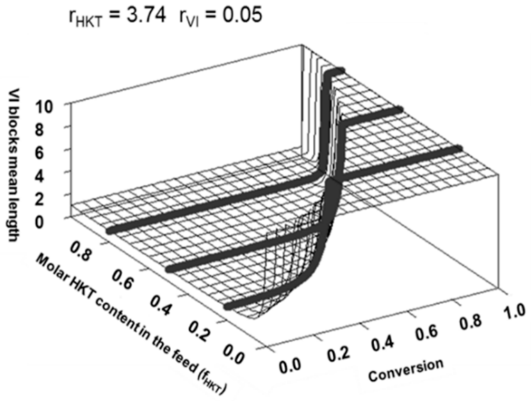

Figure S3. Surface diagrams representing the variation of the instantaneous molar fraction of HKT in the copolymer.

Preparation and characterization of NPs

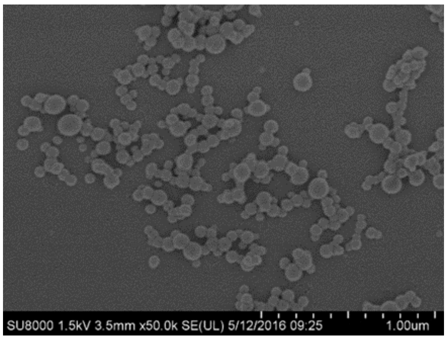

Figure S4. Scanning Electron Microscopy micrograph showing morphology of KT NPs.

Dexamethasone encapsulation

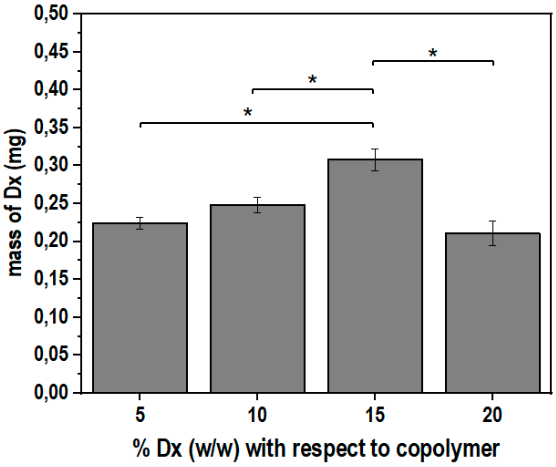

Figure S5. Mass of dexamethasone encapsulated measured by HPLC for each initial %Dx (w/w) with respect to poly(HKT-*co*-VI) copolymer. Results represent mean ± SD of two independent experiment, n = 2, * $p \leq 0.05$.

RT-qPCR

Table S1. RT-qPCR primer list.

Gene Symbol	Official Full Name	Species	Forward (5′-3′)	Reverse (5′-3′)
Il23a	Interleukin-23 subunit alpha	mouse	AATAATGCTATGGCTGTTGC	CTTAGTAGATTCATATGTCCCG
Tnfa	Tumor necrosis factor	mouse	CTATGTCTCAGCCTCTTCTC	CATTTGGGAACTTCTCATCC
Il12b	Interleukin-12 subunit beta	mouse	CATCAGGGACATCATCAAAC	CTCTGTCTCCTTCATCTTTTC
Vegfa	Vascular endothelial growth factor A	mouse	TAGAGTACATCTTCAAGCCG	TCTTTCTTTGGTCTGCATTC
Tgfb1	Transforming growth factor beta-1 proprotein	mouse	GGATACCAACTATTGCTTCAG	TGTCCAGGCTCCAAATATAG
Il10	Interleukin-10	mouse	CAGGACTTTAAGGGTTACTTG	ATTTTCACAGGGGAGAAATC
Actb	Actin, beta	mouse	GATGTATGAAGGCTTTGGTC	TGTGCACTTTTATTGGTCTC

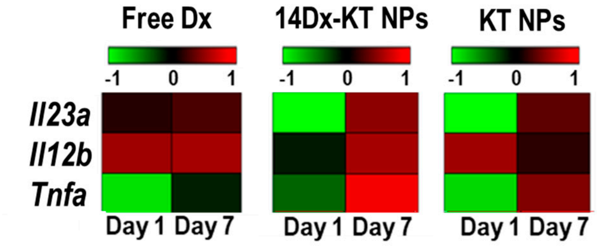

Figure S6. Quantitative real-time PCR data. Heat map of gene transcript levels of M1 markers in non-LPS activated samples (CM) treated with free dexamethasone (free Dx, 5.1 μM, red), Dx-loaded ketoprofen-bearing NPs (14Dx-KT NPs, 5.1μM Dx and 0.045 mg/mL NPs, white) and unloaded ketoprofen-bearing NPs (KT NPs, 0.045 mg/mL, black) for 1 day (plain) and 7 days (dashed) Results are expressed relative to the corresponding level of expression of each transcript in the untreated sample.

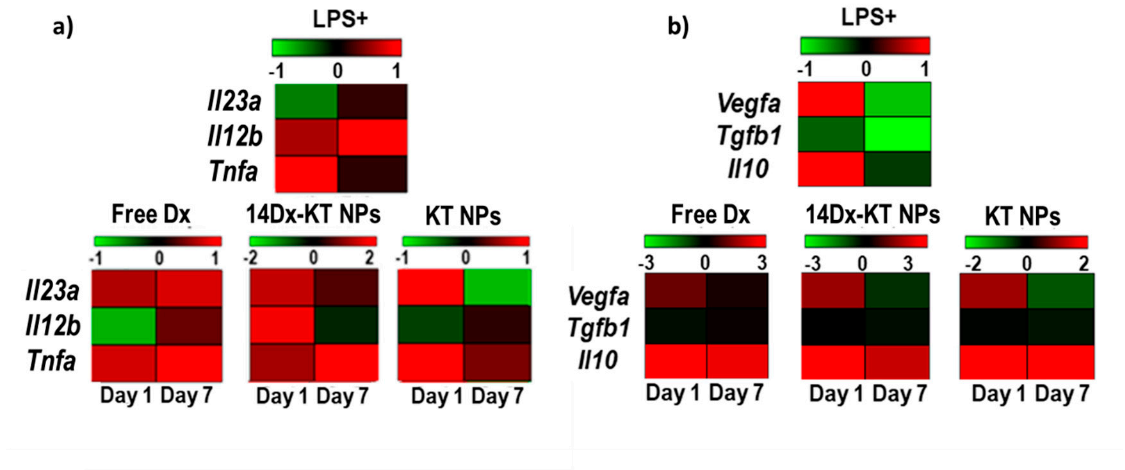

Figure S7. Quantitative real-time PCR data. Heat maps of gene transcript levels of **a)** M1 markers and, **b)** M2 markers in inflamed samples (500 ng/mL of LPS) treated with culture media (LPS+, blue), free dexamethasone (free Dx, 5.1 μM, red), Dx-loaded ketoprofen-bearing NPs (14Dx-KT NPs, 5.1μM Dx and 0.045 mg/mL NPs, white) and unloaded ketoprofen-bearing NPs (KT NPs, 0.045 mg/mL, black) for 1 day (plain) or 7 days (dashed). Results are expressed relative to the corresponding level of expression of each transcript in the untreated sample (CM).

Table S2. Quantitative real-time PCR data. Gene transcript levels of M1 markers under normal cellular conditions (CM) and of M1 and M2 markers under inflammatory conditions (LPS+), treated with free dexamethasone (free Dx, 5.1 μM), Dx-loaded ketoprofen-bearing NPs (14Dx-KT NPs, 5.1μM Dx and 0.045 mg/mL NPs) and unloaded ketoprofen-bearing NPs (KT NPs, 0.045 mg/mL) for 1 day or 7 days. Data are presented as mean log2 variation and standard deviation compared to untreated cells in 2 independent experiments, each quantified in triplicate.

M1 markers (CM)						
**Genes**	**Free Dx**		**14Dx-KT NPs**		**KT NPs**	
	**Day 1**	**Day 7**	**Day 1**	**Day 7**	**Day 1**	**Day 7**
Il23a	1.13 ± 0.12	0.15 ± 0.07	-0.22 ± 0.06	0.24 ± 0.04	-0.08 ± 0.04	0.24 ± 0.04
Il12b	0.55 ± 0.23	0.33 ± 0.09	-0.19 ± 0.09	0.27 ± 0.08	0.55 ± 0.08	0.24 ± 0.09
Tnfa	-0.19 ± 0.02	0.08 ± 0.01	0.17 ± 0.06	0.27 ± 0.11	-0.47 ± 0.05	0.12 ± 0.04

M1 markers (LPS+)								
**Genes**	**CP (+LPS)**		**Free Dx**		**14Dx-KT NPs**		**KT NPs**	
	**Day 1**	**Day 7**	**Day 1**	**Day 7**	**Day 1**	**Day 7**	**Day 1**	**Day 7**
Il23a	-0.50 ± 0.06	0.19 ± 0.03	0.61 ± 0.04	0.74 ± 0.29	1.34 ± 0.25	0.56 ± 0.26	1.14 ± 0.30	-0.74 ± 0.08
Il12b	0.59 ± 0.06	3.43 ± 0.10	-0.69 ± 0.15	0.36 ± 0.09	1.69 ± 0.30	-0.33 ± 0.23	-0.27 ± 0.11	0.18 ± 0.10

Tnfa	3.59 ± 0.11	0.18 ± 0.02	0.72 ± 0.11	0.90 ± 0.02	1.11 ± 0.13	2.70 ± 0.50	0.99 ± 0.23	0.42 ± 0.04
**M2 markers (LPS+)**								
Vegfa	1.02 ± 0.01	-0.77 ± 0.02	1.10 ± 0.01	0.35 ± 0.01	1.58 ± 0.04	-0.61 ± 0.01	1.09 ± 0.003	-0.70 ± 0.01
Tgfb1	-0.39 ± 0.01	-2.00 ± 0.05	-0.27 ± 0.02	0.22 ± 0.01	0.02 ± 0.00	-0.28 ± 0.00	-0.09 ± 0.00	-0.22 ± 0.02
Il10	2.51 ± 0.02	-0.25 ± 0.03	3.21 ± 0.01	2.47 ± 0.03	3.38 ± 0.01	2.08 ± 0.00	2.74 ± 0.02	2.17 ± 0.03

References

1. Aguilar, M.R.; Gallardo, A.; Fernández, M.d.M.; Román, J.S. In Situ Quantitative 1H NMR Monitoring of Monomer Consumption: A Simple and Fast Way of Estimating Reactivity Ratios. *Macromolecules* **2002**, *35*, 2036-2041, doi:10.1021/ma0106907.
2. Pekel, N.; Şahiner, N.; Güven, O.; Rzaev, Z.M.O. Synthesis and characterization of N-vinylimidazole–ethyl methacrylate copolymers and determination of monomer reactivity ratios. *European Polymer Journal* **2001**, *37*, 2443-2451, doi:https://doi.org/10.1016/S0014-3057(01)00124-0.
3. Andersson Trojer, M.; Hansson, Ö.; Öhrström, L.; Idström, A.; Nyden, M. Vinylimidazole copolymers: Coordination chemistry, solubility, and cross-linking as function of Cu2+ and Zn2+ complexation. *Colloid and Polymer Science* **2011**, *289*, 1361-1372, doi:10.1007/s00396-011-2461-5.
